# lncRNA187415.1 silence in BCAMs ameliorated breast cancer progression by blocking C/EBPβ‐lncRNA187415.1‐CISH axis and reversing pro‐tumor characteristic of BCAMs

**DOI:** 10.1002/ctm2.407

**Published:** 2021-05-06

**Authors:** Jiali Li, Lin Xia, Fang Liu, Yu Tian, Rong Chen, Zhaoliang Su

**Affiliations:** ^1^ International Genome Center Jiangsu University Zenjiang China; ^2^ Department of Immunology Jiangsu University Zhenjiang China

Dear Editor,

Recently, we demonstrated that lncRNA187415.1 knockdown in breast cancer‐associated macrophages (BCAMs) could change their phenotype and function of BCAMs, benefit the BCAMs inducing breast cancer cells apoptosis and inhibit their invasion. LncRNA187415.1 maintained BCAMs function in local microenvironment by inhibiting CISH mRNA degradation; however, transcription factor C/EBPβ regulated transcription of lncRNA187415.1 via binding promoter region of –1500∼–2000 bp of lncRNA187415.1.

As our previous research about lncRNA profiles of BCAMs using lncRNAs microarray showed that^1^ lncRNA187415.1 was the most significantly changed gene. The data were further confirmed in BCAMs isolated from breast cancer bearing mice in vitro (Figures 1A and 1B). To further investigate the biological role of lncRNA187415.1, its expression in BCAMs was successfully silenced by siRNAs (Figure 1C). LncRNA187415.1 silence in BCAMs significantly inhibited the proliferation, migration, and invasion of 4T1 cells (Figures 1D and 1E). Conversely, lncRNA187415.1 silence in BCAMs resulted in a significantly increase of the apoptosis in 4T1 cells (Figure 1F). To further confirm the similar effects also exist in vivo, the jetPEI‐Man/ si‐lncRNA187415.1 complex was used to specifically downregulate the lncRNA187415.1 in BCAMs of breast cancer bearing mice. A dramatic reduction of tumor volumes and tumor weight were observed in si‐lncRNA187415.1 group compared to si‐NC (Figures 1G and 1H). These results clearly demonstrated that lncRNA187415.1 knockdown target on BCAMs significantly ameliorated breast cancer progression.

**FIGURE 1:**
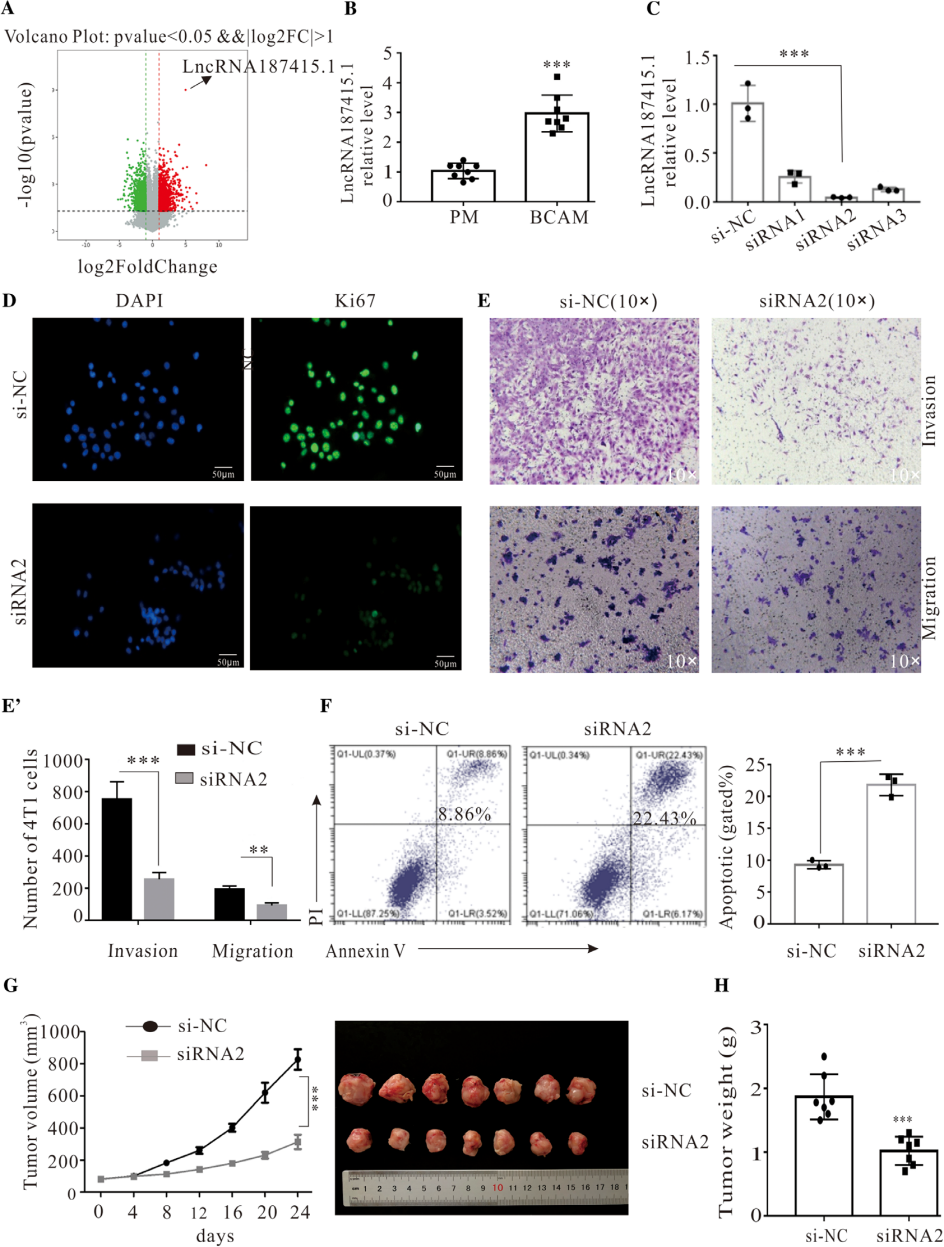
LncRNA187415.1 silence in BCAMs ameliorated breast cancer progression. (A) Volcano plot of lncRNAs gene profiles results in BCAMs. The expression of lncRNAs in macrophages and BCAMs was detected by lncRNAs microarray technology. Upregulation (red), downregulation (green), and no‐significance (gray). (B) qPCR analysis for lncRNA187415.1 expression in BCAMs from breast tumors (*n* = 8). (C) siRNA knockdown efficiency. siRNAs (50 nM) were transiently transfected into BCAMs, after 48 h, BCAMs were harvested for qPCR analysis (*n* = 3). (D) Representative images of 4T1 are stained by Ki67 (green) and DAPI (blue). LncRNA187415.1‐downregulated BCAMs were co‐cultured with 4T1 for 48 h. The proliferation of 4T1 was evaluated by Ki‐67/DAPI staining. Data were statistically analyzed based on three independent experiments. (E) Invasion and migration ability of 4T1 cells co‐cultured with lncRNA187415.1 downregulated BCAMs. Data represent means ± SD from three independent experiments. (F) Apoptosis of 4T1 cells was analyzed by Annexin V and PI staining. Note that 4T1 cells were co‐cultured with LncRNA187415.1‐downregulated BCAMs for 48 h; the apoptosis of 4T1 cells was analyzed by flow cytometry (*n* = 3). (G) Tumor volume. Note that 4T1 tumor‐bearing mice were given jetPEI‐Man/si‐NC complex or jetPEI‐Man/siRNA2 complex. Tumor volume measurement was performed by digital caliper at the indicated days (*n* = 7). (H) Tumor weight. Tumors were resected at day 24, and tumor weight was measured (*n* = 7). All data are shown as means ± SD. ^**^ *p *< 0.01, ^***^ *p *< 0.01 Abbreviation: PM, peritoneal macrophage.

To determine the effect of lncRNA187415.1 on the phenotype and function of BCAMs, lncRNA187415.1 was silenced in BCAMs with siRNA. After lncRNA187415.1 silence, CD86 and MHC II were significantly increased, conversely, CD206 was downregulated, LPS‐treated macrophages (pro‐inflammatory type) and IL‐4‐treated macrophages (anti‐inflammatory type) were used as positive or negative control, respectively (Figure 2A). The qPCR and ELISA also indicated that the expression of *Tnfα* and *Il‐1β* mRNA as well as their proteins levels were also increased after lncRNA187415.1 silence, and the mRNA and protein expression of *Il‐4*, *Il ‐10* were decreased in si‐lncRNA187415.1 group (Figures 2B and 2D). Furthermore, the protein level of Arginase 1 (Arg1) decreased and iNOS increased in si‐lncRNA187415.1 group (Figure 3C). Taken together, these results suggested that lncRNA187415.1 silence could reprogram the phenotype of BCAMs.

**FIGURE 2:**
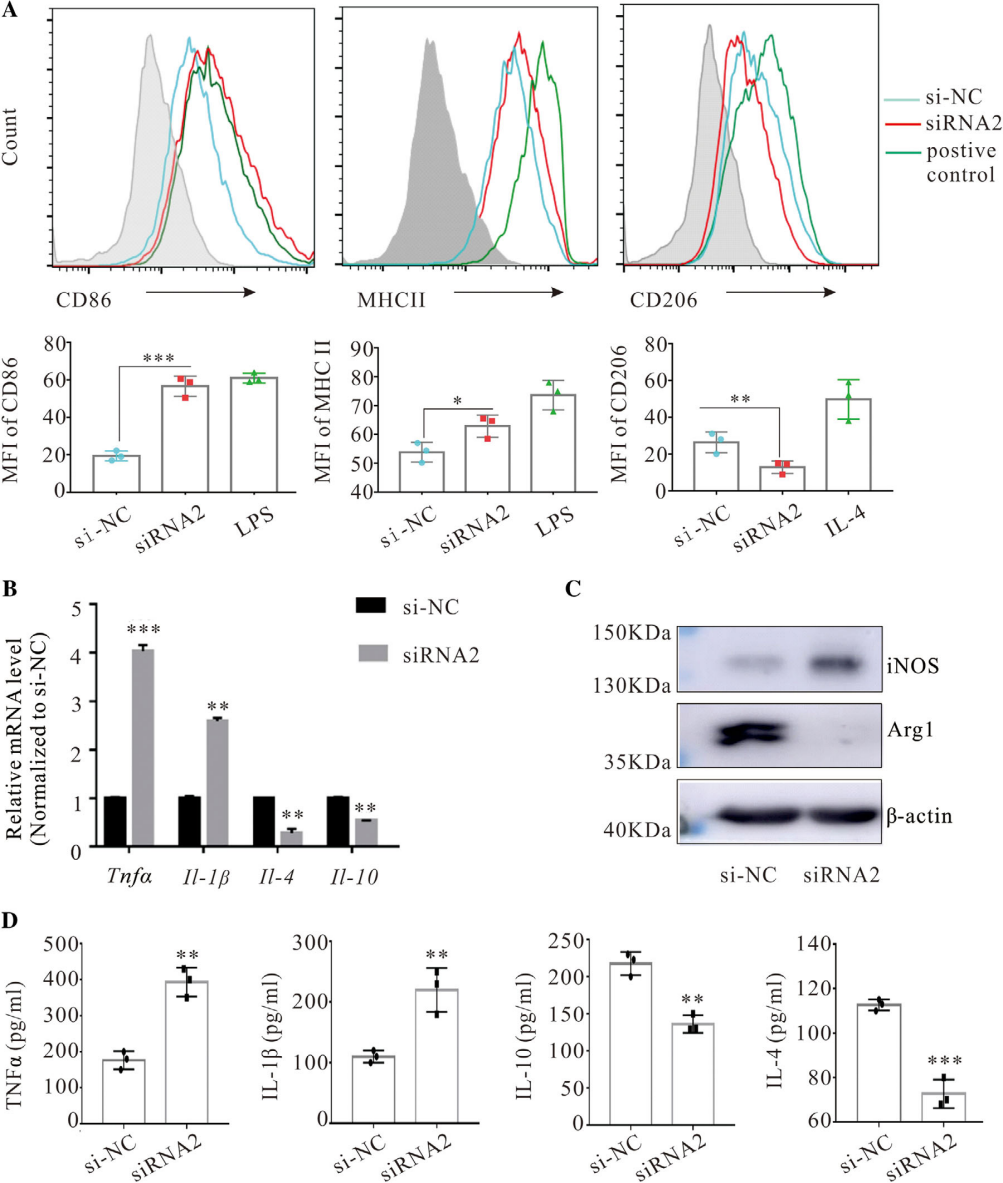
LncRNA187415.1 silence reprogrammed the phenotype and function of BCAMs. BCAMs with siRNA2 or si‐NC transfected for 48 h, and then BCAMs and supernatant were harvested. (A) Flow cytometry analysis for surface protein markers (CD86 and MHCII, and CD206). (B) qPCR analyzed the mRNA level of cytokines. (C) Western blotting analyzed the protein levels of iNOS and Arg1. (D) ELISA analyzed the secretion of cytokines. Data represent means ± SD from three independent experiments. ^*^ *p* < 0.05, ^**^ *p* < 0.01 and ^***^ *p* < 0.001

**FIGURE 3:**
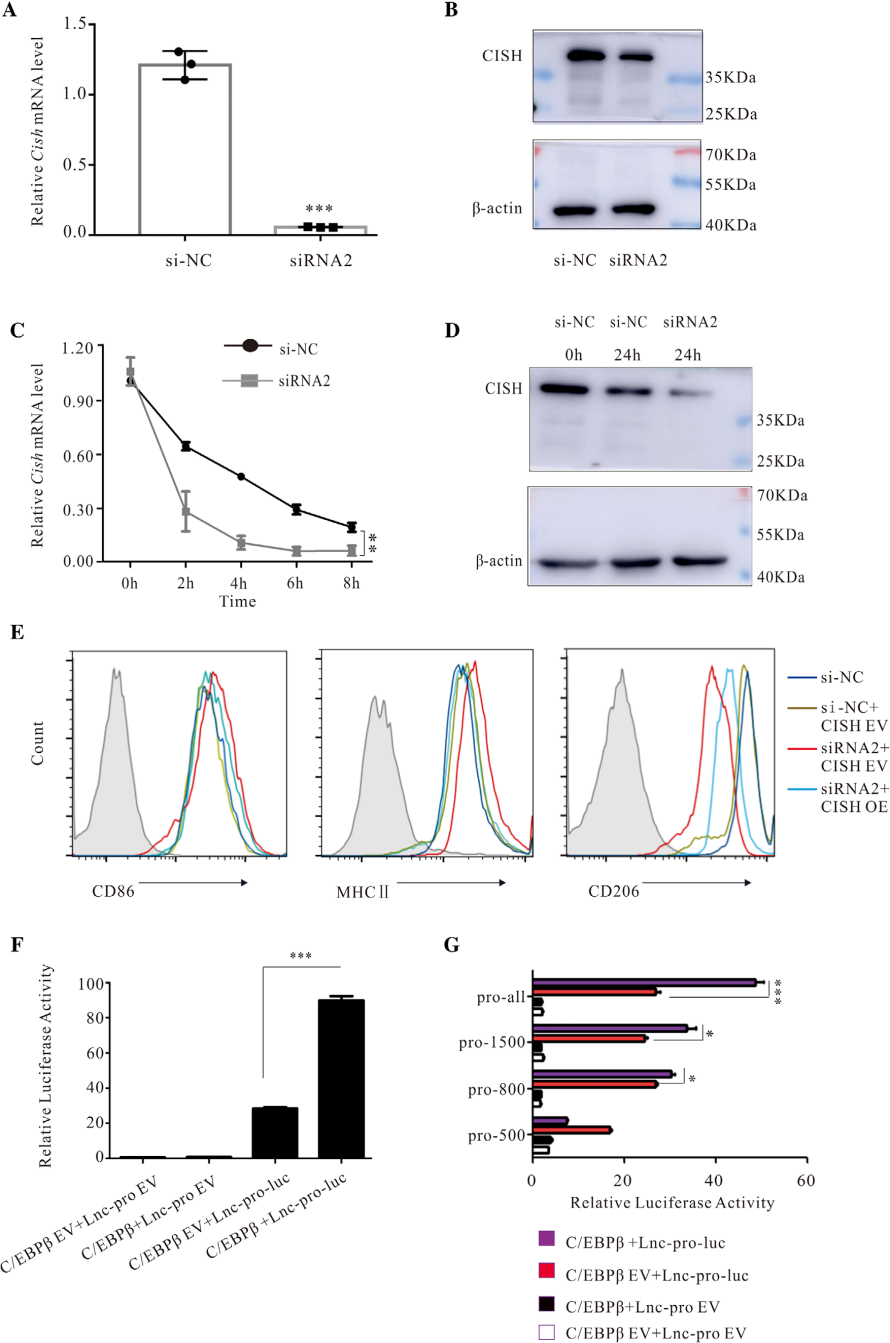
LncRNA187415.1 activated by C/EBPβ maintained the pro‐tumor characteristics of BCAMs by facilitating CISH mRNA stability. (A and B) qPCR analyzed *Cish* mRNA, and protein levels were detected by qPCR and Western blotting. Data are shown as means ± SD. (C and D) BCAMs with siRNA2 or si‐NC transfected for 48 h, and then treated by 5 μg/ml actinomycin D for indicated time. The mRNA stability was evaluated using mRNA decay curves, and protein level was analyzed by Western blotting. Data were analyzed based on three independent experiments. (E) BCAMs with siRNA2 or si‐NC co‐cultured for 48 h, and then CISH overexpressed plasmid was transfected for the other 48 h. Flow cytometry analysis for surface protein markers. (F) Luciferase assay of 293T cells after co‐transfection of lncRNA187415.1 promotor plasmid (Lnc‐pro‐luc) and C/EBPβ overexpressing plasmid, *n* = 3. (G) Luciferase assay of 293T cells after co‐transfection of lncRNA187415.1 promotor plasmids with different length of promotor and C/EBPβ overexpressing, *n* = 3. ^***^ *p* < 0.001, ^*^ *p* < 0.05. pro‐all: sequence ‐2 kb ∼ ±0 bp, including all potential binding sites; pro‐1500: sequence ‐1.5 kb ∼ +0 bp, including five potential binding sites; pro‐800: sequence –800 bp ∼ ±0 bp, including three potential binding sites; pro‐500: sequence ‐500 bp ∼ ±0 bp, including one potential binding sites. All data were analyzed based on three independent experiments. ^**^ *p* < 0.01, ^***^ *p* < 0.001

As the results above showed that lncRNA187415.1 silence in BCAMs could benefit the shift of BCAMs phenotype and function from anti‐inflammatory to pro‐inflammatory. To further clarify the mechanism of lncRNA187415.1 maintaining BCAMs showing anti‐inflammatory phenotype in local microenvironment, downstream target genes related to lncRNA187415.1 in BCAMs were analyzed by RNA microarray. The results indicated that CISH is the potential target gene (Log_2_FC = 8.07) (Figure S1A and Table S1). Previous data also indicate that CISH is related to the polarization of macrophages.^2^ To confirm whether lncRNA187415.1 could promote the expression of CISH, firstly, lncRNA187415.1 was silence in BCAMs, lncRNA187415.1 silence significantly reduced the expression of CISH mRNA and protein level (Figures 3A and 3B). To further confirm how lncRNA187415.1 regulated CISH, actinomycin D, an RNA polymerase inhibitor, was employed. After actinomycin D treatment, the half‐life of *CISH* mRNA was significantly shortened in lncRNA187415.1 silence group compared with control (Figures 3C and 3D), which indicated that lncRNA187415.1 could be involved in transcription‐related processes of *CISH* and facilitate the stability of *CISH* mRNA. To further confirm, the shift of BCAMs phenotype and function caused by *CISH* downregulation induced by lncRNA187415.1 silence, overexpressed plasmid of CISH was transfected into the BCAMs following lncRNA187415.1 silence for 48 h, the results showed that expression of CD86 and MHC II decreased, conversely, CD206 increased (Figure 3E).

To clarify which molecule regulates lncRNA187415.1 in BCAMs, PROMO database (http://alggen.lsi.upc.es/home.html) was used to predict the transcription factors that bind to lncRNA187415.1 promoter region. C/EBPβ was a candidate transcription factor for lncRNA187415.1. To further investigate the regulation of C/EBPβ on lncRNA187415.1, *Cebpb* was silence in BCAMs by siRNA (Figure S1B and S1D), the expression of lncRNA187415.1 and *Cish* was significantly decreased (Figure S1C and S1E), which confirmed that C/EBPβ regulated the expression of lncRNA187415.1 as a transcript factor. Then the JASPER database (http://jaspar.genereg.net/) was used to predict eight sites that could bind to the lncRNA187415.1 promoter region as follows (Figure S1F), and sequential truncations of this lncRNA187415.1 luciferase reported were created to identify regions critical for lncRNA187415.1 transcription. Four luciferase reporter plasmids containing pro‐all (including all the sites), pro‐1500 (containing five sites), pro‐800 (containing three sites), and pro‐500 (containing one site) were constructed in order to determine the approximate binding region. The result indicated that the fewer binding sites included, the lower the luciferase activity was. Additionally, Only the pro‐1500 of the transcription factor was activated and bound to more than 30% (C/EBPβ+Lnc‐pro‐luc/C/EBPβ EV+Lnc‐pro‐luc) (Figures 3F and 3G); therefore, the C/EBPβ bound to the lncRNA187415.1 promoter region from –1.5 kb to –2 kb, and ChIP‐qPCR experiment was performed to verify the binding sites (Figure S1G).

Our data clearly demonstrated that C/EBPβ‐LncRNA187415.1‐CISH maintained the pro‐tumor characteristics of BCAMs and contributed to breast cancer progression. LncRNA187415.1 silence in BCAMs inhibited breast cancer cells proliferation, migration, and invasion, promoted apoptosis, and ameliorated breast cancer progression. Our results shed a light on immunotherapy of breast cancer targeting BCAMs.

## CONFLICT OF INTEREST

The authors have declared that no conflict of interest exists.

## FUNDING INFORMATION

This work was supported by Primary Research and Development Plan of Jiangsu Province (grant number: BE2019700), Jiangsu Province “333” project (grant number: BRA2018016), Six talent peaks project in Jiangsu Province (grant number: 2019‐WSN‐122), Projects of International Cooperation from Jiangsu (grant number: BX2019100), and International cooperation and exchange from Zhenjiang (grant number: GJ2020010).

## ETHICS APPROVAL AND CONSENT TO PARTICIPATE

All animal procedures were in compliance with the Guide for the Care and Use of Laboratory Animals (NIH, 76 FR 91 [May 11, 2011]) and were approved by the Animal Care and Use committee of Jiangsu University.

## CONSENT FOR PUBLICATION

All authors approve to submit it as the present style.

## DATA AVAILABILITY STATEMENT

All the data were shown in the manuscript and are available from the correspondence author upon request.

## Supporting information




**Figure S1 (A)** Volcano plot of downstream target genes related to lncRNA187415.1 in BCAMs. Upregulation (red), downregulation (green), and no‐significance (gray). BCAMs with siRNA2 or si‐NC transfected for 48 h, and then BCAMs were harvested. (**B and D**) Knockdown efficiency of CEBP/β siRNA was evaluated by qPCR and Western blotting analysis. siRNAs (50 nM) were transiently transfected into BCAMs, after 48 h, BCAMs were harvested for qPCR analysis. (**C)** qPCR analysis for the levels of lncRNA187415.1 and *CISH* mRNA. Data are shown as means ± SD (*n* = 3). (**E)** Western blotting analysis for the protein level of CISH. (**F)** Predict the binding sites of C/EBPβ to lncRNA187415.1 promoter region using Jaspar database. (**G)** Primers for seven binding sites were designed for ChIP‐qPCR to verify the luciferase assay results.

Table S1 The potential targets of lncRNA187415.1
