# Supplementary figures and images for: lncRNA187415.1 silence in BCAMs ameliorated breast cancer progression by blocking C/EBPβ‐lncRNA187415.1‐CISH axis and reversing pro‐tumor characteristic of BCAMs
Source: Clin Transl Med. 2021 May 6;11(5):e407. doi: 10.1002/ctm2.407 (PMC8102855; doi:10.1002/ctm2.407)

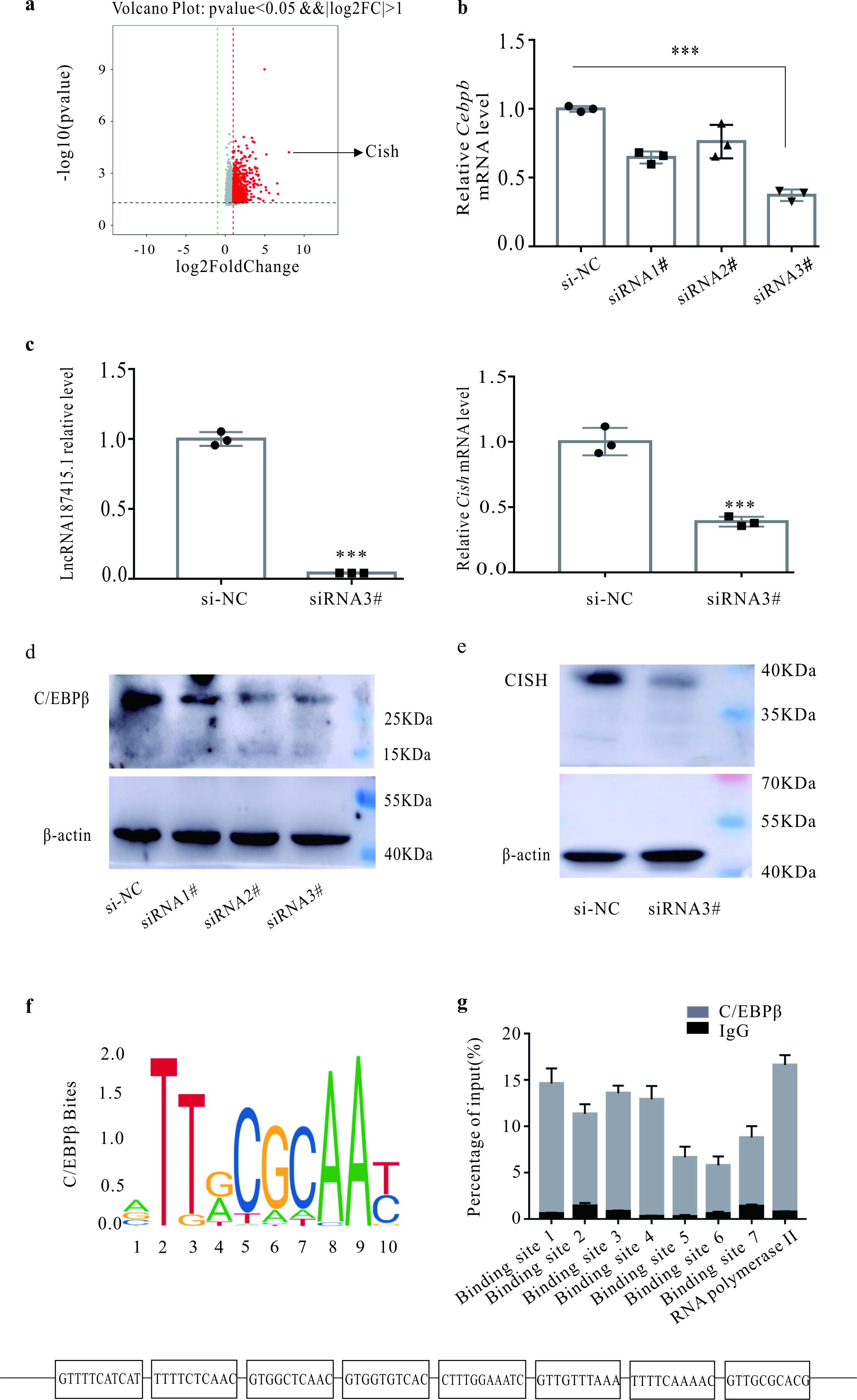

Supplement: Supplementary file 1 — Figure S1 (A) Volcano plot of downstream target genes related to lncRNA187415.1 in BCAMs. Upregulation (red), downregulation (green), and no‐significance (gray). BCAMs with siRNA2 or si‐NC transfected for 48 h, and then BCAMs were harvested. (B and D) Knockdown efficiency of CEBP/β siRNA was evaluated by qPCR and Western blotting analysis. siRNAs (50 nM) were transiently transfected into BCAMs, after 48 h, BCAMs were harvested for qPCR analysis. (C) qPCR analysis for the levels of lncRNA187415.1 and CISH mRNA. Data are shown as means ± SD (n = 3). (E) Western blotting analysis for the protein level of CISH. (F) Predict the binding sites of C/EBPβ to lncRNA187415.1 promoter region using Jaspar database. (G) Primers for seven binding sites were designed for ChIP‐qPCR to verify the luciferase assay results. [file CTM2-11-e407-s002.jpg]
